# Trisomy silencing by XIST: translational prospects and challenges

**DOI:** 10.1007/s00439-024-02651-8

**Published:** 2024-03-09

**Authors:** Khusali Gupta, Jan T. Czerminski, Jeanne B. Lawrence

**Affiliations:** 1 Department of Neurology, University of Massachusetts Chan Medical School, Worcester, MA 01655, USA; 2 Department of Pediatrics, University of Massachusetts Chan Medical School, Worcester, MA 01655, USA; 3 Medical Scientist Training Program, University of Massachusetts Chan Medical School, Worcester, MA 01655, USA

## Abstract

XIST RNA is heavily studied for its role in fundamental epigenetics and X-chromosome inactivation; however, the translational potential of this singular RNA has been much less explored. This article combines elements of a review on XIST biology with our perspective on the translational prospects and challenges of XIST transgenics. We first briefly review aspects of XIST RNA basic biology that are key to its translational relevance, and then discuss recent efforts to develop translational utility of XIST for chromosome dosage disorders, particularly Down syndrome (DS). Remarkably, it was shown in vitro that expression of an XIST transgene inserted into one chromosome 21 can comprehensively silence that chromosome and “dosage compensate” Trisomy 21, the cause of DS. Here we summarize recent findings and discuss potential paths whereby ability to induce “trisomy silencing” can advance translational research for new therapeutic strategies. Despite its common nature, the underlying biology for various aspects of DS, including cell types and pathways impacted (and when), is poorly understood. Recent studies show that an inducible iPSC system to dosage-correct chromosome 21 can provide a powerful approach to unravel the cells and pathways *directly* impacted, and the developmental timing, information key to design pharmacotherapeutics. In addition, we discuss prospects of a more far-reaching and challenging possibility that XIST itself could be developed into a therapeutic agent, for targeted cellular “chromosome therapy”. A few rare case studies of imbalanced X;autosome translocations indicate that natural XIST can rescue an otherwise lethal trisomy. The potential efficacy of XIST transgenes later in development faces substantial biological and technical challenges, although recent findings are encouraging, and technology is rapidly evolving. Hence, it is compelling to consider the transformative possibility that XIST-mediated chromosome therapy may ultimately be developed, for specific pathologies seen in DS, or other duplication disorders.

## Introduction

We will begin with an overview of basic biology of XIST RNA to provide a foundation for considering the translational potential of this unique RNA which silences a whole chromosome. The fundamental biology of XIST RNA and female X-chromosome inactivation has been very heavily studied and reviewed elsewhere [e.g. (Balaton et al. 2018; Brockdorff et al. 2020; Creamer and Lawrence 2017; Jacobson et al. 2022)], whereas the focus here will be on how this natural mechanism can be relevant to the problem of chromosomal imbalances, particularly Down Syndrome (DS). Given hundreds of studies on X-chromosome dosage compensation by mouse Xist or human XIST RNA, we regret we could not cite all the relevant references, so in several cases we cite recent reviews. We also note that much of our knowledge of how this RNA functions is based on mouse ES cells or mice, which is largely paralleled in human, but there are some known (and potentially unknown) differences.

DS is the most common chromosomal trisomy occurring in ~ 1/750 live births in the US. However, collectively chromosomal abnormalities, including many rare small chromosomal imbalances, are even more common, occurring in ~ 1/150 newborns (Hsu 1992). A much larger fraction of spontaneous miscarriages is caused by various chromosomal imbalances. Hence, chromosomal imbalances comprise a large part of the human genetic burden yet have remained largely outside the hopeful advances in genetics and targeted biomedical therapies. As we will highlight further below, a few early case studies in rare patients with unbalanced X;autosome (X;A) translocations indicated to us that XIST RNA has some ability to silence autosomes sufficiently to rescue otherwise lethal trisomies (Allderdice et al. 1978; Hall et al. 2002; Leisti et al. 1975). This prompted work to directly test the extent to which a targeted *XIST* transgene could transcriptionally silence autosomal genes on one of the three copies of chromosome 21 in patient-derived induced pluripotent stem cells (iPSCs). Surprisingly, induced XIST RNA resulted in essentially complete silencing of genes across that autosome. Having demonstrated dosage compensation of chromosomal abnormality in vitro, how can this remarkable ability of XIST forge new paths to advance translational research and therapeutic strategies for this or potentially other chromosomal dosage disorders? We will outline three general pathways where use of XIST transgenes can help identify new therapeutic strategies, either as a novel investigative strategy, or, possibly, as a therapeutic agent. After summarizing key aspects of XIST RNA biology and evidence from rare cases of X;A translocations, we will then summarize findings showing the value of an inducible iPSC system to “silence trisomy”. This provides a powerful and needed experimental system for translational research, and also addresses biological questions pivotal to whether XIST transgenics could be developed into a transformative epi/genetic therapy for chromosomal disorders.

### XIST RNA: nature’s remarkable solution to silence a whole chromosome

**Fig. 1:**
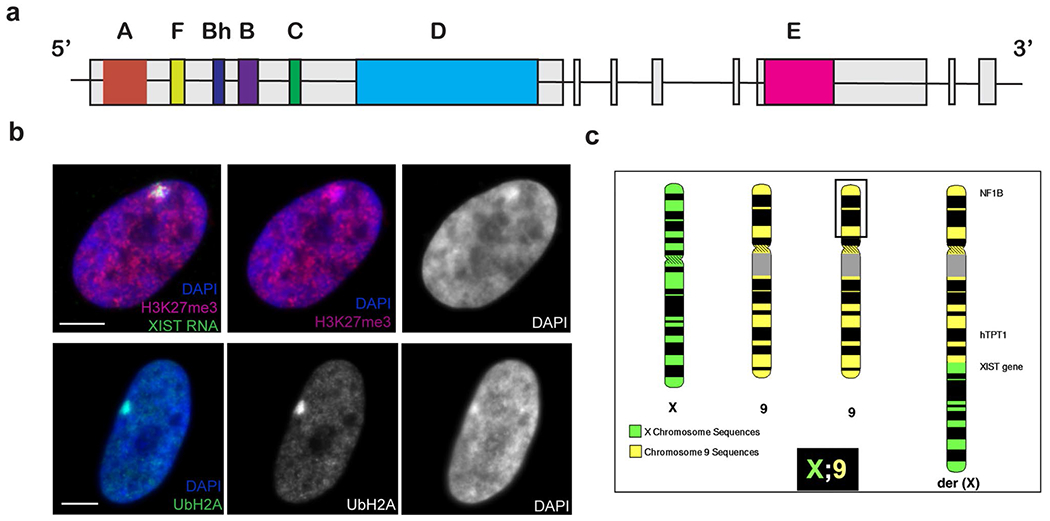
XIST and X-inactivation. **a** Map of the human X-inactivespecific transcript (XIST) gene indicating the positions of seven distinct tandem repeat regions (colored boxes), most of which are conserved and thought important to XIST RNA function. Grey boxes represent the eight exons of XIST. **b** The XIST RNA territory localizes over the condensed DNA on the inactive X-chromosome (Barr body), and is marked by repressive epigenetic modifications, shown in human Tig-1 fibroblasts. Top panel: Staining for trimethyl histone H3K27 (H3K27me3, red) and XIST RNA (green) on the Barr body, (DAPI, blue, shown in black and white). Bottom panel: Staining for ubiquitinated histone H2A (green) over the Barr body (DAPI, blue). Scale bars, 5 μm. **c** Ideogram of an unbalanced X:9 translocation of a woman carrying an extra copy of chromosome 9 translocated to the long arm of X-chromosome. The black box marks the distal region on one chromosome 9 p-arm that was missing

In mammals, the difference in X-linked gene dosage between males and females is dosage compensated by random inactivation of one of two female X-chromosomes. XIST (X-inactive-specific transcript) is a long (14–17 kb) non-coding RNA (lncRNA) that contains several regions of conserved tandem repeats (Brown et al. 1992) (Fig. 1a). Key to understanding XIST RNA function was the finding that, in interphase nuclei of human female cells, stable XIST transcripts spread and tightly localize in *cis* across the whole inactive X-chromosome territory, marked by the condensed Barr Body (Clemson et al. 1996). Early in embryogenesis, XIST RNA coats and stably silences one X-chromosome by triggering a cascade of repressive chromatin modifications, whereas the XIST gene on the active X becomes silenced by CpG methylation (Norris et al. 1994). However, some genes escape silencing, particularly in the pseudoautosomal region (which is also present on the Y chromosome) (Posynick and Brown 2019). Importantly, because XIST recruits redundant layers of numerous repressive biochemical changes (e.g. to histones and DNA methylation), it establishes a stable silent state which is then inherited by all subsequent cells (Brockdorff et al. 2020; Jacobson et al. 2022; Loda et al. 2022) (Fig. 1b). While much research has focused on XIST RNA recruitment of histone modifications needed for local gene silencing, recent studies from our lab indicate that XIST RNA embeds as part of chromosome structure (Creamer et al. 2021) and likely also acts on large-scale structural elements to condense the chromosome (Valledor et al. 2023).

An aspect of XIST RNA biology that may prove advantageous to its translational utility is the redundant stability of epigenetic silencing the RNA induces. Although XIST RNA continues to be expressed in somatic cells throughout life, studies of both mouse Xist and human XIST have shown that the heterochromatic state and gene silencing remains almost fully stable even if the RNA is no longer expressed or removed (Brown and Willard 1994; Csankovszki et al. 2001; Jiang et al. 2013). Interestingly, B-cells appear to be an exception where XIST RNA briefly de-localizes and a number of genes are re-activated (Wang et al. 2016; Yu et al. 2021).

As shown in Fig. 1, the long XIST transcript (14–17 kb) has 7 regions of distinct tandem repeats that are largely conserved in mouse and thought important for XIST function. As recently reviewed elsewhere (Boeren and Gribnau 2021; Brockdorff et al. 2020; Loda and Heard 2019; Raposo et al. 2021) much XIST research currently focuses on attempts to delineate the function of these different repeat regions, primarily by studying the impact of deleting certain regions on gene silencing and various chromatin modifications. The most studied repeat is the small 5’ A-repeat domain, which consists of ~ 9 copies of ~ 46 bp repeats that form hairpin loops. It was initially reported that deletion of the 5’ fragment from Xist caused loss of silencing function in mouse ES cells (Wutz et al. 2002) and this has been confirmed in mice (Hoki et al. 2009; Colognori et al. 2020; Royce-Tolland et al. 2010) as well as human cell systems (Chow et al. 2007).

It has been shown in several studies (Brockdorff et al. 2020) that the A-repeat domain contributes to Xist RNA’s silencing function via interaction with SPEN to recruit histone deacetylases. However, how other repeat regions contribute to Xist/XIST RNA function is much less clear, but this is under investigation, primarily for mouse Xist, but some studies are also investigating transgenic human XIST (Dixon-McDougall and Brown 2022; Valledor et al. 2023). While X-inactivation is known to involve Polycomb complexes PRC1 and PRC2, there has not been consensus regarding the roles of the different repeats or order of events, reflecting the complexity of interactions and steps involved. Some evidence indicates that the B and C repeats of XIST/Xist interact with the RNA binding protein hnRNPK, which then recruits PRC1 and PRC2 and their corresponding repressive marks, H2AK119ub and H3K27me3, respectively (Brockdorff et al. 2020). Other repeat domains may be important for the RNA’s chromosomal association and spread; for example, some evidence indicates repeat E interacts with CIZ1 (CDKN1A Zinc finger protein 1) and that this is important for localization of XIST/Xist RNA to the chromosome (Ridings-Figueroa et al. 2017; Sunwoo et al. 2017). We anticipate that as more is learned about the contributions of different XIST domains, this will be important to maximize the utility and translational relevance of XIST transgenics, as will become clearer in the discussion below.

### Rescue of rare patient trisomies suggested XIST’s translational potential

Many hundreds of basic biology studies were prompted by discovery of XIST; however, we are at an early stage of exploring the translational potential of this paradigm shifting non-coding RNA. Some studies explore ways to *circumvent* the outcome of XIST function (the stable silent state) for X-linked diseases in which it would be beneficial to reactivate a silenced normal allele in female cells, since females are essentially mosaic for expression of the normal versus mutant allele (Migeon 2020; Wang et al. 2021). For example, in Rett syndrome, mutation of the X-linked MeCP2 gene causes embryonic lethality in males but also causes a devastating neurological disorder in young girls (Shah and Bird 2017). Hence, several studies are seeking ways to circumvent the stability of XIST-mediated silencing of the normal MeCP2 allele to develop therapeutic strategies for Rett syndrome (Grimm and Lee 2022; Leko et al. 2018; Przanowski et al. 2018; Qian et al. 2023). Since XIST RNA induces multi-layered repressive modifications that maintain the silent state, broader efforts at “epigenome editing” may also advance the feasibility of reactivating silenced X-chromosome genes (Gjaltema and Rots 2020; Holtzman and Gersbach 2018).

A distinct question is whether XIST function can be harnessed to silence a trisomic chromosome, particularly an autosome, and early insights could be gleaned from clinical human genetics, as summarized in Table 1. It is known that women with trisomy X have two silenced X-chromosomes, and most lead normal lives without apparent impact of harboring an extra chromosome in all cells (Tartaglia et al. 2010). This demonstrates two points critical for the translational potential of XIST: the compatibility of two XIST-expressing chromosomes with normal life, and that the physical presence of millions of extra base pairs of DNA is not disruptive nor pathogenic, as has been proposed [e.g., (Plona et al. 2016)]. Rather, the transcriptional silencing of trisomic genes by two-fold XIST RNA coating two Barr bodies is benign, and indeed lifesaving in that it rescues an otherwise lethal X-chromosome trisomy.

A key question then is whether XIST RNA, if expressed from an autosome, could similarly localize across and silence that autosome *in cis*. Earlier studies of patients with balanced X;autosome translocations showed the prevalent outcome is that the translocation is *not* silenced and the intact X is non-randomly silenced (Mattei et al. 1982; Schmidt and Du Sart 1992; Summitt et al. 1978). While there was some evidence of partial autosomal silencing in some human X;autosome translocations, in most cases little to no autosomal silencing was seen. The generally limited silencing of autosomal material may seem to indicate that specific X-enriched sequences are required. However, an alternative explanation is that silencing autosomal material (in balanced translocations) would be selected against, since it would create a deleterious functional monosomy. To avoid this issue of selection against autosomal silencing, which also impacts random autosomal Xist transgenes in mESCs, we searched for case reports of rare X-autosome translocations involving a trisomic autosome which normally would be an embryonic lethal. We identified two reports of individual patients with surprisingly mild phenotypes for which we were able to obtain lymphoblastoid cell lines, to examine XIST RNA (Allderdice et al. 1978).

As summarized in Table 1, one such case involves a boy with Klinefelter syndrome who had a few minor dysmorphic features despite the presence of nearly an entire extra copy of chromosome 14 translocated to part of the X. Trisomy 14 is generally incompatible with life, thus this indicated there must be substantial silencing of the translocated autosome (Allderdice et al. 1978). Another case involved a woman with a third copy of most of chromosome 9 translocated to the long arm of the X-chromosome (Leisti et al. 1975) (Fig. 1c). This patient did exhibit morphological abnormalities and learning disabilities, but considering her karyotype, the effects were unexpectedly mild. Since these patients were described before discovery of XIST, subsequent examination of cultured cells from these patients demonstrated that XIST RNA from the translocated X region did indeed spread across much of the autosomal chromatin, along with hallmarks of inactivation (Hall et al. 2002). Another case was later reported of a girl with mild developmental delay and dysmorphic features caused by trisomy 15 with an X;15 translocation (Stankiewicz et al. 2006). Since trisomy for 15q causes severe cognitive disability and dysmorphic features (Kristoffersson and Bergwall 1984; Pedersen 1976), this patient’s milder phenotype implied that a large portion of 15q is likely silenced. Together, these cases motivated an experimental effort to examine the full potential of XIST to silence an autosomal trisomy.

### XIST fully silences trisomy 21 in vitro: potential pathways to therapies

**Fig. 2:**
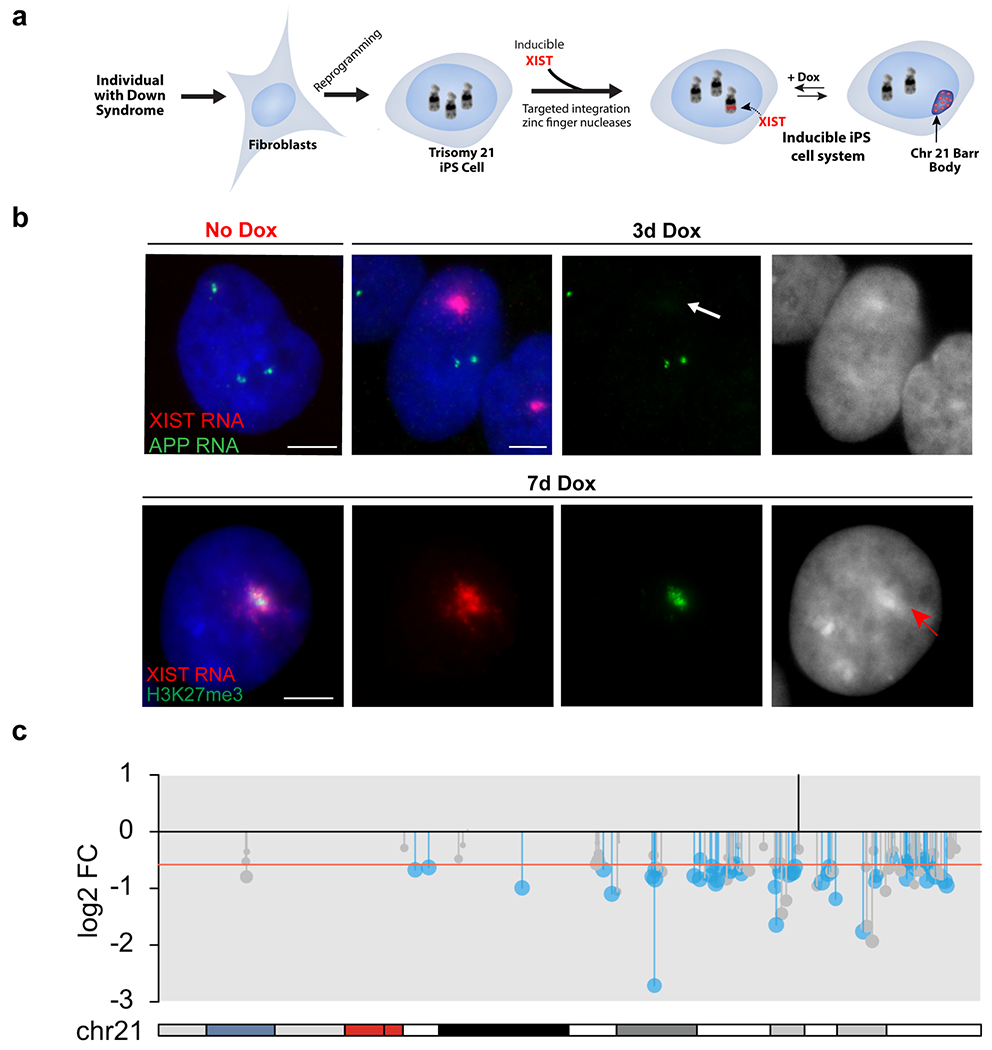
Trisomy 21 chromosome silencing. **a** Diagram depicting the development of the inducible system to silence one of the three chromosome 21s in DS patient-derived iPS cells, as initially demonstrated by (Jiang et al. 2013). **b** Illustration of chromosome 21 gene silencing by XIST RNA. Top panel: Detection of APP RNA transcription foci from three alleles (green) are seen in iPS cell nucleus without Dox induction of XIST (left image). Right panel: Three days after Dox induction a large XIST RNA accumulation (red) has silenced one of the three APP alleles (green). Individual channel showing APP RNA signals with arrow showing absence of a third APP RNA signal where XIST cloud is located and DAPI (blue, shown in black and white) signal showing brighter focus of compacted DNA coincident with XIST RNA cloud. Bottom panel: Induction of XIST RNA with 7 days of Dox produces epigenetic changes on chromosome 21 as shown by H3K27me3 (green) co-localized with XIST RNA (red) and DAPI DNA (blue). B&W image of DAPI staining shows brighter focus of compact DNA (red arrow) coincident with XIST RNA and H3K27me3 signals. Scale bars, 5 μm. **c** RNAseq affirms that XIST RNA induction decreases expression of genes across the silenced chromosome 21, shown in DS endothelial cells. Red line indicates theoretical one-third reduction in mRNA levels, and vertical black line marks site of XIST insertion. Significant genes are in blue (FDR < 0.05), with other expressed genes in gray (Moon and Lawrence 2022)

Given the medical and societal importance of Down syndrome, our priority became to test the concept of “trisomy 21 silencing”, done in induced pluripotent stem cells, the natural cell context in which XIST expression and the chromosome silencing process begins. XIST RNA localizes and functions *in cis*, and thus testing autosomal silencing required targeted insertion of the large XIST transgene into a Chr21 (Jiang et al. 2013). At the time (before the CRISPR era), it was dubious that even this first step would be technically possible. However, ultimately a very large inducible XIST transgene (21 kb construct carrying 14 kb XIST cDNA) was successfully targeted using ZFNs (zinc finger nucleases) into one chromosome 21 (Fig. 2a). Analysis of parallel cell cultures with and without doxycycline-induced XIST expression showed that all heterochromatin marks typical of the inactivated X-chromosome (Xi) were induced on a “chromosome 21 Barr body” and microarrays showed chromosome-wide gene repression (Fig. 2b). Several subsequent RNAseq studies have shown that chromosome 21 silencing is remarkably robust and complete (Fig. 2c) (Czerminski and Lawrence 2020; Moon and Lawrence 2022). Surprisingly, results show little evidence of “escape genes” on Chr21, in contrast to numerous genes that escape silencing on the Xi. This indicates that escape from silencing is likely due to enrichment of certain “escape” sequences on parts of the X-chromosome rather than lack of specific “silencing sequences”, consistent with previous suggestions [e.g. (Cotton et al. 2014; McNeil et al. 2006)]. The distribution of Alu and LINE1 repeats may influence heterochromatin formation (Lawrence et al. 2024), but interspersed repeats are generally abundant across most human chromosomes. The demonstrated silencing of hundreds of genes across chromosome 21 provided proof of a novel concept: the natural mechanism of chromosome silencing could be redirected to “dosage compensate” a trisomic autosome.

**Fig. 3:**
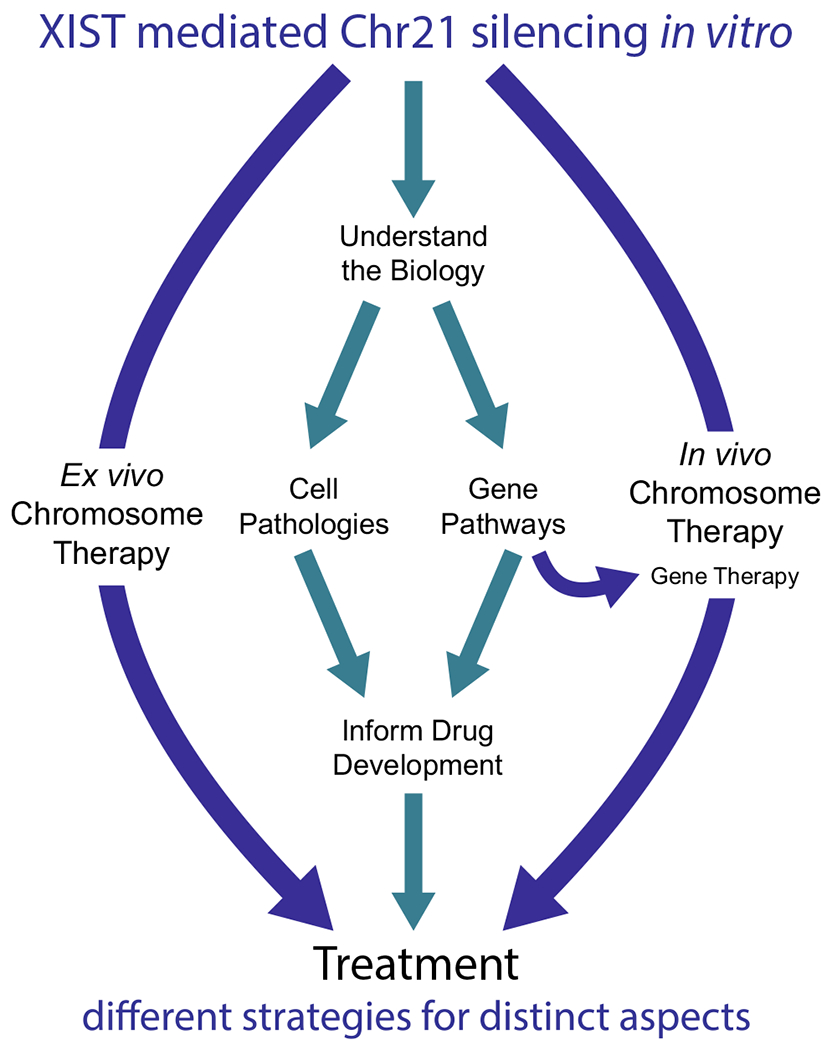
Pathways to treatment of genetic diseases. Potential pathways whereby the ability of XIST to silence trisomy can advance translational research and therapeutic strategies for systems impacted in Down syndrome. The middle pathway utilizes inducible “trisomy silencing” as a powerful experimental approach to investigate the underlying biology of human trisomy 21, information key to design of traditional pharmacotherapeutics, or potentially gene therapies. A more formidable but potentially transformative path is the possibility that an XIST transgene itself could be developed as an agent for “chromosome therapy”, which could potentially involve ex vivo or in vivo delivery. Because this approach addresses the root cause of various cell pathologies, over-expression of chromosome 21 genes, it circumvents the need to unravel and address the complexity of pathways and genes involved. While chromosome therapy is a compelling concept, and recent findings are encouraging, this nonetheless is a farreaching and challenging prospect

The demonstration that XIST could “silence Trisomy 21” in vitro provided a pivotal revelation: the problem of over-expression for hundreds of genes could be reduced to manipulation of a single gene, XIST. This breakthrough concept may have had some immediate impact by challenging the then-common presumption that chromosomal imbalances are beyond the reach of genetics and biomedical therapies. But how might the ability to silence Trisomy 21 help advance translational research for DS? Fig. 3 summarizes different pathways by which XIST-based transgenics could lead to translational advances for medical challenges in DS, or potentially other chromosomal disorders. We first discuss the middle pathway which uses trisomy silencing as a powerful and experimental approach to investigate the underlying biology of human DS to advance target identification for more traditional therapeutics. We will then discuss the more far-reaching possibility that XIST itself (or XIST derivative transgenes), could be developed into a therapeutic agent.

The biology of trisomy 21 is complex, and the medical challenges faced by millions of people worldwide with DS can vary substantially between individuals. In addition to craniofacial abnormalities, trisomy 21 consistently confers some degree of cognitive disability, which may be progressive, but this can vary widely between people. Children with DS are typically sociable and well-loved members of families. But trisomy 21 also confers increased risks for over a dozen potential co-occurring conditions, which are also seen in the non-DS population (Antonarakis et al. 2020; Hendrix et al. 2021). These include highly elevated risks of congenital heart defects and leukemia in children, and frequent pulmonary hypertension, diabetes, arthritis, hypothyroidism and Hirschsprung Disorder in adults. Importantly, it is now known that trisomy 21 almost inevitably causes early-onset Alzheimer Disease (linked to trisomy for the APP gene), and thus afflicting individuals as early as in their 40s and 50s. Recent studies highlight the importance of inflammation and immune dysfunction in DS due to triplication of several clustered interferon receptor genes on Chr21, which a recent study in mice suggests underlies to many major aspects of the syndrome (Waugh et al. 2023).

**Fig. 4:**
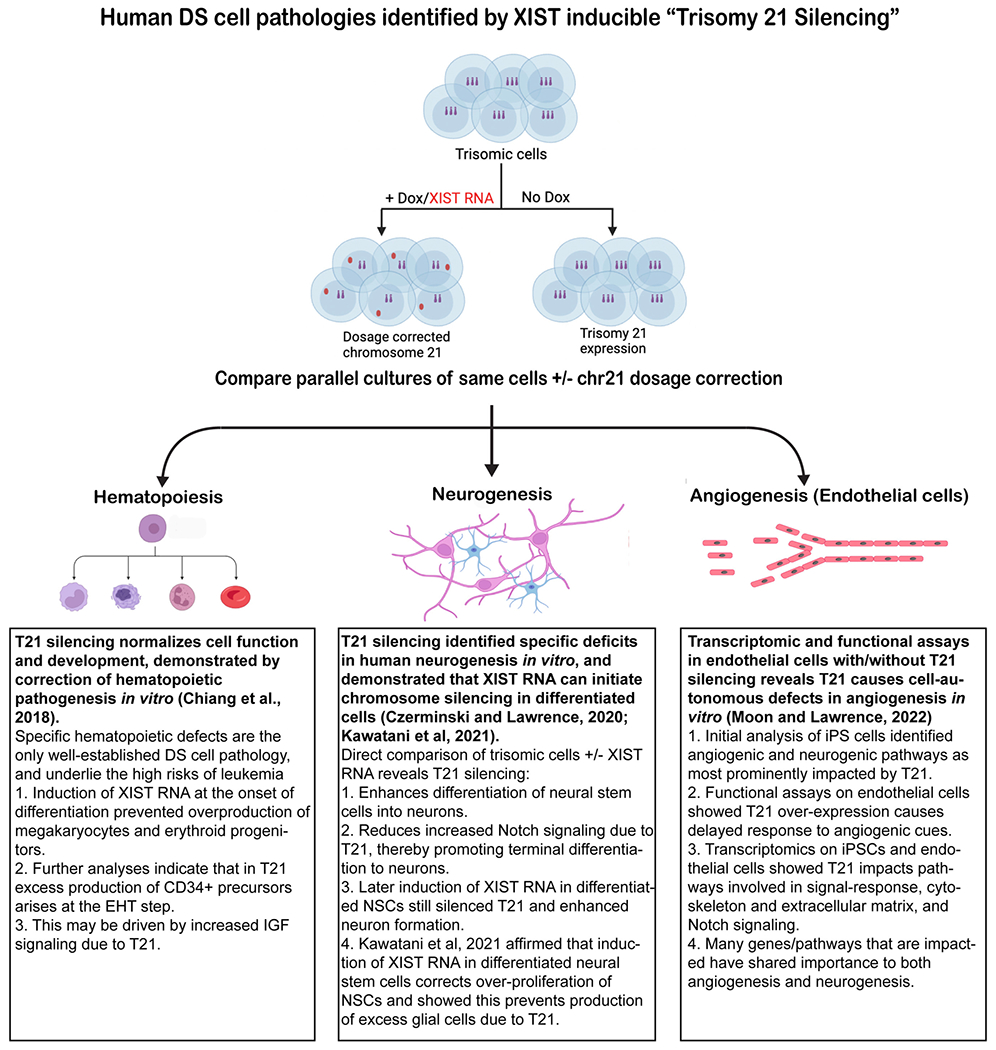
Identifying direct effects of Trisomy 21 on human cell types and gene pathways. Surprisingly little is known as to what human cell types are directly (or indirectly) impacted by trisomy 21, nor is it known when deficits arise. A system to induce silencing of one Chr21 (by XIST RNA) in DS patient-derived stem cells provides a tightly controlled approach to identify if there are direct effects of chr21 over-expression on various human cell types, and also provides a novel means to test when changes arise and may be reversible. Note, this approach compares identical populations of cells with and without dox induction and, therefore, avoids confounding variation that often arises between isogenic iPS cell lines. Figures created with Biorender.com

Unlike most single-gene disorders, the underlying biology of DS, including the cell types and pathways impacted, is poorly understood. The field has relied heavily on mouse models carrying partial trisomies, thus better ways to study the human developmental cell biology are needed, as an inducible XIST iPSC system can provide (see Figs. 2 and 4). The best established human cell phenotype is the over-production of erythrocytes and megakaryocytes during perinatal hematopoiesis, which greatly increases pre-leukemia and leukemia (AMKL and AML) risks for DS children (de Castro et al. 2021). To test whether transcriptional silencing of one Chr21 can prevent this known DS cell phenotype, (Chiang et al. 2018) tested this for hematopoiesis and affirmed that induced XIST expression during in vitro differentiation of iPSCs indeed corrects this known hematopoietic pathogenesis. Given this validation, we recently used this approach to study if and how trisomy 21 impacts other developmental cell types, and to identify impacted pathways, in studies briefly summarized in Fig. 4. A study of neurogenesis in vitro (Czerminski and Lawrence 2020) revealed that silencing one chromosome 21 reproducibly enhances the differentiation of neural stem cells to neurons; parallel RNAseq analysis implicated certain non-chromosome 21 pathways and genes (notably involving Notch signaling). Another study of trisomy silencing, that began with unbiased analysis of iPSCs, led to a study of endothelial cells which uncovered a cell autonomous dampening in the response of trisomic endothelia to angiogenic signals (Moon and Lawrence 2022) (Fig. 4). This study further implicated specific genes/pathways that are common to and important in both angiogenesis and neurogenesis.

It is important to note that this “trisomy silencing” approach examines parallel cultures of the same cells, with and without induced XIST; this is advantageous because it circumvents the variability that commonly arises between isogenic iPSC lines, which our studies suggest can confound results [e.g. (Czerminski et al. 2022)]. While this work is at an early stage and primarily from our lab, this approach using inducible XIST has begun to be adapted in other labs, including in a published study of trisomy 21 silencing in astroglia cells (Kawatani et al. 2021) and another bioRxiv paper examining effects on cell stress or proliferation of neural and glia cells (Bansal et al. 2023). Hence, this inducible trisomy silencing approach can overcome certain weaknesses of relying on mouse studies or cell line comparisons and complement other efforts to identify targets for drug therapies (Antonarakis et al. 2020).

### XIST can initiate silencing in post-differentiation neural cells

As noted above, XIST expression and chromosome silencing naturally begins in the inner cell mass, and it was previously thought that pluripotency was required for Xist to function for chromosome silencing with possible exception of hematopoietic cells (Savarese et al. 2006; Wutz and Jaenisch 2000). In any case, the ability to produce various cell types and organoids from iPSCs with/without dosage-correction is itself very valuable to dissect the biology of trisomy 21. However, if initiating XIST RNA expression in post-differentiation cells could still induce the cascade of changes that silence one Chr21, the translational potential of XIST would be greatly augmented. In the translocation patients described above, XIST (on the trisomic translocation chromosome) was present since conception with RNA expression beginning in pluripotent cells. To expand the power of XIST as an experimental system, and for any application of chromosome therapy to be biologically feasible (in utero or postnatally), XIST must initiate silencing in differentiated cells. Fortunately, when we directly tested this in iPSC-derived neural stem cells, results demonstrated that chromosome silencing indeed still takes place in differentiated cells, albeit the full silencing process occurs more slowly (~ 2 weeks versus ~ 4 days) (Czerminski and Lawrence 2020). Subsequently, another published study (Kawatani et al. 2021) generated XIST-inducible trisomic iPSCs and affirmed that induction of XIST in differentiated NSCs or astroglia progenitor cells results in effective repression of chromosome 21 genes. Due to technical silencing of the inducible promoter used, we could not induce the XIST promoter to begin expression in post-mitotic neurons, but XIST expression induced in differentiated NSCs shortly before terminal differentiation resulted in neurons with robust XIST expression and localization, accompanied by repression of genes across chromosome 21. Importantly, these neurons maintain RNA expression and repressed chromosome 21 genes at a similar level to the cells that remained as NSCs, indicating neurons support XIST RNA function in chromosome silencing. In light of this technical complication, we advised other labs to use a different promoter, and this enabled them to initiate XIST expression after the terminal differentiation step, as recently reported in bioRxiv (Bansal et al. 2023).

While tests of XIST RNA function will be extended to other cell types, the confirmed competence of differentiated cells to enact chromosome silencing was a vital step forward and encourages that other cells may well retain substantial “chromatin plasticity”. As further discussed below, the demonstrated competence of differentiated neural cells to support XIST RNA function substantially expands the potential utility of XIST for translational epigenetics.

### Timing of developmental impacts and cell phenotype correction

It was important to show that XIST RNA can function in post-differentiation cells, but a distinct and critical question concerns when during post or prenatal life do specific impacts arise? For example, Alzheimer Disease clearly occurs later in adulthood, but the developmental timing of cognitive deficits is more difficult to gauge. The ability to dosage-correct the root cause of a given pathology (trisomy 21) in post-differentiation cells provides a means to determine when a specific cellular phenotype arises, and when it remains reversible or amenable to therapeutic correction. In addition to showing that XIST RNA function does not require pluripotency factors, Czerminski and Lawrence (2020) also showed that cortical neurogenesis is similarly enhanced if XIST expression is first induced to begin the chromosome silencing process *later* in differentiated neural cells. Importantly, whether dox induction of XIST RNA was begun in iPSCs or two weeks after neural differentiation, a similar increase in the terminal differentiation of NSCs to neurons was seen. Other findings also indicated that it was a delay in this terminal differentiation step that was “rescued” by trisomy silencing. RNAseq showed that trisomy 21 causes increased Notch signaling which is known to promote continued cycling of NSCs, thereby delaying terminal differentiation. Continued cycling of neural progenitor cells is known to favor their differentiation to glial cells rather than neurons, and increased glia has been reported in human DS. Relevant to this and the findings by Czerminski and Lawrence (2020), Kawatani et al. (2021) showed that induction of XIST RNA in differentiated NSCs prevented overproduction of trisomic astroglial cells.

These studies illustrate the power of this inducible system to investigate when a given cellular impact of trisomy 21 arises and when it remains amenable to improvement if the root cause (trisomic expression) is corrected. This question of timing is key to designing rational therapeutic strategies of any type and will differ for the multiple systems impacted in DS. To detail the various systems impacted in DS is beyond the scope of this review, but we will briefly highlight a few considerations. Clearly some important aspects of this syndrome, such as early-onset Alzheimer Disease and pulmonary hypertension, develop later in adulthood. Many adults and families adjust well to the challenges of living with intellectual disability, but a chief concern becomes the effects of Alzheimer dementia which afflicts 80% or more of DS adults, about 20 years earlier than the non-DS population (Fortea et al. 2021). In contrast, congenital heart defects occur very early and are largely in place by 8 weeks of gestation (the earliest detection of most trisomy 21 cases). By 8 weeks, neurogenesis has already begun, however it will continue until the third trimester (Bystron et al. 2006; Malik et al. 2013), and substantial brain development and maturation continues after birth. For example, neurogenesis continues in the cerebellum, which is smaller at birth and demonstrates decreased growth in infants and children with trisomy 21; reduced granule cell proliferation in the cerebellum was reported to be largely corrected by a single injection of a Sonic hedgehog (Shh) agonist in one day old trisomic mice (Das et al. 2013). Other work has indicated trisomy causes a deficit in myelination; a process that continues past adolescence (Olmos-Serrano et al. 2016). Additionally, Trisomy 21 may not just impact cell development, but ongoing cellular functions, such as ion channels or inhibitory/excitatory imbalances in the brain (Bartesaghi 2023). While some neurodevelopmental impacts of trisomy are surely present at birth, in our view it remains an important but unresolved question as to the extent of cognitive disability present at birth, since infants and children may often score more mildly or moderately impacted than adults. In a small fraction of cases there is marked cognitive regression in younger adults, and it remains to be determined how the increased inflammation and immune dysfunctions that are prevalent in DS contribute to overall function and quality of life.

Considerably more work must be done, but the finding that there is not a hard biological barrier to the efficacy of XIST RNA when first expressed in differentiated cells expands the potential applications. Further work in mouse models of trisomy and advanced human cellular models, such as cerebral organoids (Czerminski et al. 2022), are promising avenues to further examine the potential and developmental limits for dosage compensation to illuminate or even ameliorate specific cellular and system phenotypes.

### Technological challenges to chromosome therapy

The concept of XIST-mediated chromosome therapy reduces the problem of hundreds of trisomic genes to the “simple” challenge of targeted insertion of one gene. This paradigm shift now makes the revolutionary technical progress in gene therapy for single-gene disorders relevant to DS (and potentially other duplication disorders). However, since XIST RNA works *in cis*, “chromosome therapy” with *XIST* would not only require efficient delivery to cells, but the targeted insertion of the XIST transgene into the chromosome, and, in fact, into one of three chromosomes. Hence, even for a single gene, these are significant technical hurdles to overcome. We note that prior to our successful efforts to target an XIST transgene into the human chromosome 21 in human iPSCs (Jiang et al. 2013), we were advised the technology could not facilitate targeted insertion of such a large transgene. The extent and nature of technical challenges for XIST-mediated chromosome therapy will differ substantially depending on the tissues targeted and delivery strategies pursued. In vivo delivery and targeted insertion of an XIST transgene is clearly more technically challenging, whereas ex vivo delivery to potentially therapeutic cells is more feasible, and closer to what has been demonstrated in iPSC-derived hematopoietic cells (Chiang et al. 2018). Stem cell therapies are being actively pursued for various cell types, including neural stem and progenitor cells, as well as gene therapies in hematopoietic stem cells, or mesenchymal stem cells (Gowing et al. 2017; Morgan et al. 2017)]. We note that high risks of leukemia are not the only impact of trisomy 21 on the hematopoietic system; there are typically broader impacts on blood and immune systems, underlying the chronic high inflammation and contributing to other co-morbidities.

Fortunately, great strides in genetic engineering are rapidly being made, justifying optimism that many technical hurdles can be overcome. The easily programmable CRISPR-Cas9 system has been a prolific area of research (Dai et al. 2016) which uses targeted double stranded breaks repaired by cellular pathways of homology directed repair (HDR) or non-homology end joining (NHEJ). Precise nuclease variants have evolved to reduce off-target DNA cleavages, increase specificity, and enhance delivery of Cas9 components into the cell (Lino et al. 2018). In addition, newer genome editing methods that do not rely on DSBs but use catalytically impaired nucleases fused with reverse transcriptase have been developed, such as prime editors and its variants. Tremendous progress is being made to increase efficiency and delivery of gene editing components in vitro and in vivo. Even allele specific genome editing is becoming more feasible with these approaches, including the use of site-specific integrases to enhance targeted insertion of transgenes in both dividing and non-dividing cells (Chen and Liu 2023). Since the three chromosome 21 homologs in DS are typically all genetically distinct (due to non-disjunction in Meiosis I), targeting to a polymorphism present on just one Chr21 is certainly feasible.

Gene delivery techniques in recent years have largely focused on viral vectors which can provide specificity for specific organs or cell types (Demirci et al. 2022; Khirallah et al. 2023; Wang et al. 2019; Yu et al. 2023), but other non-viral approaches (such as lipid nanoparticles) are also progressing rapidly (Zhu et al. 2023). Many labs are contributing to improvements in each technical requirement, including the efficiency, specificity and safety of each step, providing impressive progress on all aspects of the gene therapy revolution (Saha et al. 2021). However, epigenetic therapy with the *XIST* gene poses a particularly difficult problem for delivery technologies due to its large size (~ 14 kb), since AAV vectors have a cargo size limit of 4.9 kb, and large sequences are generally more difficult to manipulate for genetic engineering. This currently constitutes a major technical obstacle to the feasibility of cell delivery and targeted chromosomal insertion of the full-length XIST cDNA.

### XIST minigenes to address one major technological hurdle

While many other labs are improving genetic engineering technologies, including for targeted transgene insertion, we have focused on assessing and adapting the biological potential of XIST. To this end, we have been pursuing the possibility that novel *XIST* minigenes can be identified that retain significant functionality. Recently, Valledor et al. (2023) demonstrated the exciting finding that just the tiny (450 bp) A-repeat fragment of XIST can repress several genes in the “Down syndrome critical region” (DSCR). Although the A-repeat domain was known to be important for gene silencing, it was surprising to discover that just this small 450 bp fragment (4% of the transcript) would alone be able to repress gene transcription. The A-repeat RNA does not spread over the entire chromosome like full-length XIST, but instead forms a small dense RNA focus, which surprisingly represses several genes in that local chromosome region. While the study by Valledor et al. focused on the basic biology of XIST RNA function, this illustrates that XIST minigenes have compelling promise and likely can be developed further. Much work needs to be done to further test and build on this finding, which we are pursuing in both human cells and mouse studies. It will be important to better define the extent of the chromosome region and specific genes repressed by the A-repeat RNA, and to test whether the minigene can retain this repressive function when introduced into mice. Importantly, this also provides an inducible system to determine the impacts of repressing only genes in the region suggested to be “critical” to major DS pathologies.

Ultimately A-repeat minigenes can include other functional domains being identified (Brockdorff et al. 2020; Navarro-Cobos et al. 2023; Pintacuda et al. 2017) to inform the exciting prospect that XIST transgenes can be engineered with distinct properties, such as broad or more restricted chromosomal spread. We propose that A-repeat minigenes and their derivatives may prove valuable by extending this novel epi/genetic strategy to a host of smaller chromosomal disorders that remain a largely unaddressed category of human genetic disease.

## Conclusions

The confluence of breakthroughs in XIST RNA biology, stem cell biology, and genetic engineering encourage continued pursuit of efforts to translate the natural mechanism of chromosome silencing to the common problem of chromosomal disorders. While this is challenging, we hope that the decades of research into the unique natural phenomenon of dosage compensation will be extended to advance the inherent translational relevance of chromosome silencing to chromosomal duplication disorders.

## Figures and Tables

**Table 1:** Lessons from human genetics about viable human trisomies due to silencing by XIST RNA

Condition	Karyotype; Phenotype	Take away
Trisomy X	47, XXX; Little to no phenotype (reviewed in Tartaglia et al 2010)	Presence of two XIST coated Xi or Barr bodies is largely benign Physical presence of an extra chromosome in nuclei is benign
Balanced X: autosomal translocations	46, X, (X:A). Translocation of one female X chr to an autosome with no loss/gain of genetic material, typically has no phenotype. (reviewed in Mattei et al 1982; Schmidt and Du Sart 1992)	Intact X chromosome is typically silenced Lack of silencing of the X:autosomal material is largely attributed to selection against functional monosomy
Unbalanced X: autosomal translocations where the involved autosome is trisomic	Rare case studies in which what could be a lethal trisomy produces a viable mild/moderate phenotype: 47, Y, Klinefelter male with a duplicated X:14 translocation and one X:14 is silenced. (Allderdice et al 1978; Hall et al 2002) 46, female; one intact X and most of the second X chr translocated to most of an extra chr 9. (Leisti et al 1975). XIST RNA was shown to coat much of the chr 9 material (Hall et al 2002) 46, female; one intact X and most of the second X chr translocated to most of the extra chr15. (Stankiewicz et al. 2006)	Rare case reports in which the X: autosome translocation is largely silenced thereby circumventing an otherwise lethal trisomy
